# Inverse gas chromatography in the examination of adhesion between tooth hard tissues and restorative dental materials

**DOI:** 10.1038/s41598-020-70480-6

**Published:** 2020-08-10

**Authors:** Zuzanna Buchwald, Beata Czarnecka, Adam Voelkel

**Affiliations:** 1 Institute of Chemical Technology and Engineering, Poznan University of Technology, ul. Berdychowo 4, 60-965 Poznan, Poland; 2 Department of Biomaterials and Experimental Dentistry, Poznan University of Medical Sciences, ul. Bukowska 70, 60-812 Poznan, Poland

**Keywords:** Materials science, Techniques and instrumentation

## Abstract

The adhesion is a crucial issue in the bonding of dental restorative materials to tooth hard tissues. A strong and durable bond between artificial and natural materials is responsible for the success of the restoration in the oral cavity; therefore it has to be thoroughly examined before new restorative material is introduced to the market and used clinically. Among all methods used to examine bonding strength, most of them require a large number of healthy teeth to be conducted. In this paper, the bond strength between tooth hard tissues (dentin and enamel) and an exemplary restorative composite was examined with the non-conventional method, i.e. inverse gas chromatography. Dentin and enamel from bovine teeth were separated and subjected to the standard preparation procedure using the 3-component etch-and-rinse commercial bonding system. Tissues, as well as commercial restorative composite, were examined using inverse gas chromatography. The work of adhesion between dentin/enamel and composite was calculated. Obtained results were compared with the values of shear bond strength of six configurations, i.e. etched dentin/enamel-composite, primed dentin/enamel-composite, and bonded dentin/enamel-composite. All obtained results proved that there is a correlation between the values describing bond strength obtained from inverse gas chromatography and direct mechanical tests (shear bond strength tests). It proves that inverse gas chromatography is a powerful perspective tool for the examination of bond strength between tooth hard tissues and potential dental materials without using a large number of health tooth tissues.

## Introduction

The adhesion is a crucial issue in the bonding of dental restorative materials to tooth hard tissues. This bonding occurs by mechanical and chemical interactions between the molecules of adhesive system and tooth hard tissues’ surfaces, including the inside of dentinal tubules and other irregularities present on the surface of etched dentin and enamel. A resin layer formed on the surface of these tissues connects chemically with the restoration^1^.

A strong and durable bond between artificial and natural materials is responsible for the success of the restoration in the oral cavity; therefore, it is thoroughly examined before new restorative material is introduced to the market and used clinically. There are a few methods commonly used for the examination of bond strength in the joint area of dentin/enamel—adhesive system—restorative material. Most of them consist of a direct examination of the strength of this junction. The most commonly used method for testing the bond strength between tooth hard tissues and restorative materials is shear bond strength (SBS) test and microtensile bond test^1,2^. Shear bond strength is the maximum stress that the tested material withstands under tangential load. In this test, two connected surfaces of the solids are slid, one along the other. Shear bond strength is defined as the maximum force which is registered in the moment of materials debonding^2^. However, although the most popular, this method still shows some drawbacks, in which the main is that the direction of the forces occurring during the test differs significantly from that which occurs during normal exploitation of restoratives (food chewing etc.). For this reason, tensile bond strength tests started to be used because they can evaluate the tension correctly. In this method samples of restorative materials can be placed not only on the flat surface of tissue but also in its cavity; therefore the experiment seems to be more corresponding to the natural conditions^1^. The "micro" variant of this test is also used because in that case the bonding area is small and thus, given bond strength = F/area, the bond strength would be larger and the test is more sensitive to any flaws. However, tensile and microtensile strength tests are also unable to mimic the clinical chewing force direction; therefore, artificial chewing simulators seem to be the best solution regarding the force direction. Besides these methods, there are other mechanical, as well as microscopic, methods of examination of bonding strength between tooth tissues and restoration^1^. The main disadvantage of all these direct methods is that all of them require natural healthy tooth tissues to be examined together with tested materials. These two solids have to be connected to enable direct examination. Therefore, indirect methods, not so material-consuming are being investigated.

It is well known that the adhesion between the two materials depends on their surface energy (SE)^3–5^. SE of a solid is a measure of its surface activity. It is defined as the equivalent of the surface tension of liquids, and more precisely as the energy that is needed to create (or increase) a surface unit in reversible conditions^6^. In the case of dental restorations their value of SE, as well as the value of SE of properly prepared tooth tissues will determine their bond strength^4^. SE is also related to the material’s wettability and the adhesion of bacteria, responsible for plaque formation and caries^7,8^. The most common method for determination of SE value of the solids, including dental materials, is contact angle test with the use of various solvents^3,4,7^. However, this method is susceptible to errors, and the results largely depend on the droplet size^9^ and the smoothness of the tested surface^10,11^. Inverse gas chromatography (IGC) is another method for determining the SE of solids^12–16^, and it seems much less complicated. In this variant of chromatography a tested material is placed inside the chromatographic column, while the selected test compounds with known characteristics are injected onto the column. The result of the interaction of the test compound with the examined material is a standard chromatogram with a single peak. The characteristics (retention parameter, shape) of the resulting peak illustrate the magnitude of interactions between the test compound and the examined material^12^. Retention data for the series of test compounds allow calculating the SE components. Moreover, using the IGC data it is possible to calculate the value of work of adhesion (WA) between two solid materials^5^.

IGC has been successfully used to determine the specific and dispersive components of SE of commercial glass ionomer cements^17–19^. It is also a well known method for examination of interactions between polymers^20–22^. In our previous research, IGC was used for SE values determination of bovine dentin and enamel^23^, as well as for the examination of bonding system’s effect on surface characteristics of these tissues^24^. These research proved that there is a relationship between the surface composition and changes in morphology, as well as in the values of BET specific surface area and SE parameters changes observed on the surface of crown dentin, root dentin and enamel followed by each step of tissues surface preparation (etching, priming, bonding) with the use of commercial 3-component etch-and-rinse bonding system^24^. However, there are no literature reports regarding the possible correlation between the values of WA, calculated based on IGC experiment for tooth tissues and restorative materials, and the values obtained for these tissues by the most commonly used direct method—SBS test. Therefore this study aimed to compare the values of both mentioned parameters for several combinations of dentin and enamel (following the etching, priming, and bonding) with a commercial restorative material. The research hypothesis was that there is a correlation between the values of WA calculated based on IGC and the values of SBS measured in the direct mechanical tests.

## Experimental section

### Materials

#### Tooth tissues

For all experiments, bovine incisors teeth were used. They are the most commonly used substitute for human teeth because they are morphologically and histochemically similar^25–27^. Some studies have shown that the microstructure of dentin and enamel from various mammals is very similar^28^. There are many literature reports in which bonding strength of dental fillings to human and bovine tooth hard tissues is compared. They showed no differences between the results of experiments in which hard tissues of bovine and human teeth were used^26^. Bovine teeth used in these experiments were collected from slaughterhouse within the standard procedures of meat cutting from 2 years old animals.

#### Bonding system

Preparation of the surface of bovine dentin and enamel for SBS and IGC experiments was performed with the use of commercial 3-step etch-and-rinse bonding system (OptiBond™ FL, Kerr, Italy).

#### Restorative material

Charisma (Heraeus Kulzer GmbH, Germany), shade A3 was applied in all IGC and SBS experiments as an exemplary commercial resin-based composite used in restorative dentistry. According to the manufacturer’s information, this material is composed of Bis-GMA (bisphenol-A glycidyl methacrylate) and TEGDMA (triethylene glycol dimethacrylate) monomers with binary filler, i.e. the mixture of Ba–Al–B–F–Si glass (d_50_ = 0.7 μm d_99_ < 2 μm), and pyrogenic SiO_2_ (0.01–0.07 μm), and camphorquinone photoinitiation system. Recommended curing time for 2 mm layer equals to 20 s^29^.

### Methods

#### Preparation of tooth tissues

For shear bond strength tests, 56 teeth, from which the pulp was mechanically removed, were placed in acrylic forms and polished with a carborundum gypsum model trimmer to obtain a flat, smooth surface of enamel (28 samples) and dentin (28 samples). The procedure of tooth hard tissues (dentin and enamel) preparation for IGC experiments was described in^24^. Briefly, enamel and crown dentin were separated with dental turbine drill and shredded into fragments with size from the range of 250–500 µm.

The whole surface preparation procedure was carried out according to the instruction from the manufacturer of the applied bonding system. Etching refers here to the application of etchant for 15 s and subsequent rinsing the surface with water for 15 s and air drying for 3 s. Application of primer (priming) was conducted after etching and relied on the covering of the surface with primer for 15 s with subsequent air drying for 5 s. These two steps were followed by the application of adhesive for 15 s with subsequent air drying for 3 s and photopolymerisation for 20 s. SBS testing and IGC testing were performed after each step of the presented procedure, i.e. after etching, priming, bonding. For SBS experiments after each of these steps a sample of commercial resin-based composite (RBC, Charisma) was bonded to the tissues. Thus checking the bond strength for the following connections was carried out: etched enamel-RBC, primed enamel-RBC, bonded enamel-RBC, etched dentin-RBC, primed dentin-RBC, bonded dentin-RBC. It needs to be highlighted that “primed” enamel/dentin means here that these tissues were prepared according to the first two steps of procedures mentioned above (etching and priming) before the attachment of RBC. In the same way “bonded” enamel/dentin means here that the surface of these tissues was prepared according to all preparation steps, i.e. etching, priming and bonding of bonding system before the attachment of RBC. Curing of a composite in the form of cylindrical samples (Φ 4 mm and 2 mm thick) was performed for 20 s.

LED dental curing lamp (DB 685, Coxo, China) was applied to initiate the photopolymerisation process of adhesive and composite.

#### Inverse gas chromatography (IGC)

WA was calculated for the following configurations: etched enamel-RBC, primed enamel-RBC, bonded enamel-RBC, etched dentin-RBC, primed dentin-RBC, bonded dentin-RBC, on the basis of their SE values obtained by means of IGC. SE values of tooth hard tissues were taken from our previous experiment described in our previous article^24^. SE values of Charisma were measured according to the same procedure, i.e. by placing of 0.2 g of the fragments (250–500 µm) of cured (20 s) materials in PTFE chromatographic columns (150 mm length, 4 mm internal diameter) with inert glass carrier. Nine repetitions for each material, i.e. etched dentin, primed dentin, bonded dentin, etched enamel, primed enamel, bonded enamel, and composite, were prepared and examined. SRI 8610C (SRI Instruments, USA) standard gas chromatograph was used with the flame-ionisation detector (FID). Following chromatographic conditions were set: carrier gas (helium) with flow rate equals to 15 ml·min^−1^, injector temperature: 150 °C, oven temperature: 37 °C. Experiments were conducted in the infinite dilution mode. Following substances were injected onto the columns: hexane, heptane, octane, nonane, decane (all ≥ 99%, Sigma-Aldrich, Germany), chloroform (analytical grade, Avantor, Poland), ethyl acetate (HPLC grade, Avantor, Poland). Each test solute was separately injected onto the chromatographic column at least three times. The dispersive component values of SE (γ^d^) for tissues and Charisma were calculated according to the Schultz and Lavielle procedure which has been widely described in many articles^6,12–14,19,23,24,30^. The values of the specific component of SE (γ^+^, γ^−^) for tissues and Charisma were calculated according to the Good and van Oss procedure, also widely described in previous articles^23,24,31,32^. WA was calculated as the sum of dispersive and specific components of work of adhesion:

1 $$ {\text{WA}} = {\text{WA}}^{{\text{d}}} + {\text{WA}}^{{{\text{sp}}}} \quad [{\text{mJ}}\,{\text{m}}^{ - 2} ] $$

WA^d^ and WA^sp^ are the dispersive and specific components of WA, respectively [mJ·m^−2^].

Dispersive component of WA was calculated according to the following equation:

2 $$ {\text{WA}}^{{\text{d}}} = 2 \cdot \sqrt {\upgamma _{1}^{{\text{d}}} \cdot\upgamma _{2}^{{\text{d}}} } \quad [{\text{mJ}}\,{\text{m}}^{ - 2} ] $$

where ${\upgamma }_{1}^{{\text{d}}}$ is a dispersive component of SE of the first solid (e.g. tooth tissue) [mJ·m^−2^] and ${\upgamma }_{2}^{{\text{d}}}$ is a dispersive component of SE of the second solid (e.g. RBC) [mJ·m^−2^].

The specific component of WA was calculated according to the Eq. (3):

3 $$ {\text{WA}}^{{{\text{sp}}}} = 2 \cdot \left( {\sqrt {\upgamma _{1}^{ + } \cdot\upgamma _{2}^{ - } } + \sqrt {\upgamma _{1}^{ - } \cdot\upgamma _{2}^{ + } } } \right)\quad [{\text{mJ}}\,{\text{m}}^{ - 2} ] $$

where ${\upgamma }_{1}^{ + }$ and ${\upgamma }_{1}^{ - }$ are the parameters which describe the ability of the first solid (e.g. tooth tissue) to interact as an acceptor and donor of electrons, respectively [mJ·m^−2^], while ${\upgamma }_{2}^{ + }$ and ${\upgamma }_{2}^{ - }$ are the parameters which describe the ability of the second solid (e.g. RBC) to interact as an acceptor and donor of electrons, respectively [mJ·m^−2^]^5^.

Based on SE parameters values for etched enamel, primed enamel, bonded enamel, etched dentin, primed dentin, bonded dentin and Charisma, the WA values of six possible configurations were calculated.

#### Shear bond strength (SBS) test

After bonding of RBC to the surface of dentin and enamel prepared samples were stored for 24 h and then subjected to the SBS tests. SBS tests were conducted with the use of a standard testing machine (Zwick Z010 TN ProLine, Zwick Roell, Germany). The test speed was set on 0.5 mm∙min^−1^ with the initial force equalled to 0.2 N, while the distance between the crosshead and the substrate was about 0.5 mm. The end of the test was the moment when RBC sample was detached from the surface of dentin or enamel under the influence of applied force. Shear bond strength (SBS) was calculated as the maximum stress recorded during the test.

#### Molecular modelling

Molecular modelling was conducted to optimise the structure of the main components of Charisma, i.e. Bis-GMA and TEGDMA. The optimised structures of both monomers were used to define the active sites responsible for the specific interactions of these compounds. The structures were optimised with Hartree–Fock method and 6-31G database for the molecules in the ground state. All calculations were made with Gaussian 13 software (Gaussian Inc.).

### Statistics

To establish the statistically significant differences in values of WA parameters (WA, WA^d^ and WA^sp^) and SBS between examined configurations a one-way variance analysis with post-hoc Tukey’s multiple comparison tests was applied (*p* < 0.05). For more detailed information this analysis was conducted in two test groups, i.e. for all dentin configurations and for all enamel configurations separately. No statistically significant differences were marked in the appropriate figures by the same capital letters. Moreover, the differences in WA^d^, WA^sp^, WA and SBS values between dentin and enamel on each step of preparation were tested in pairs with the use of Student’s t-tests with an independent estimation of variance (*p* < 0.05). No statistically significant differences were marked in the appropriate figures by the same lower cases. Finally, correlation Pearson’s tests were carried out to examine whether the values of WA, WA^d^, and WA^sp^ obtained from IGC experiments are correlated with the values of SBS obtained from direct mechanical tests. The level of every statistically significant (*p* < 0.05) correlation was also provided (correlation coefficient r), as well as regression equation, and coefficient of determination (r^2^).

## Results and discussion

The values of SE components obtained from IGC experiment for the studied composite are listed in Table 1.

**Table 1:** Mean values (± standard deviation) of SE components for examined composite [mJ·m^−2^].

γ^d^	γ^+^	γ^-^
35.39 (± 0.22)	7.38 (± 0.28)	14.18 (± 0.59)

**Figure 1:**
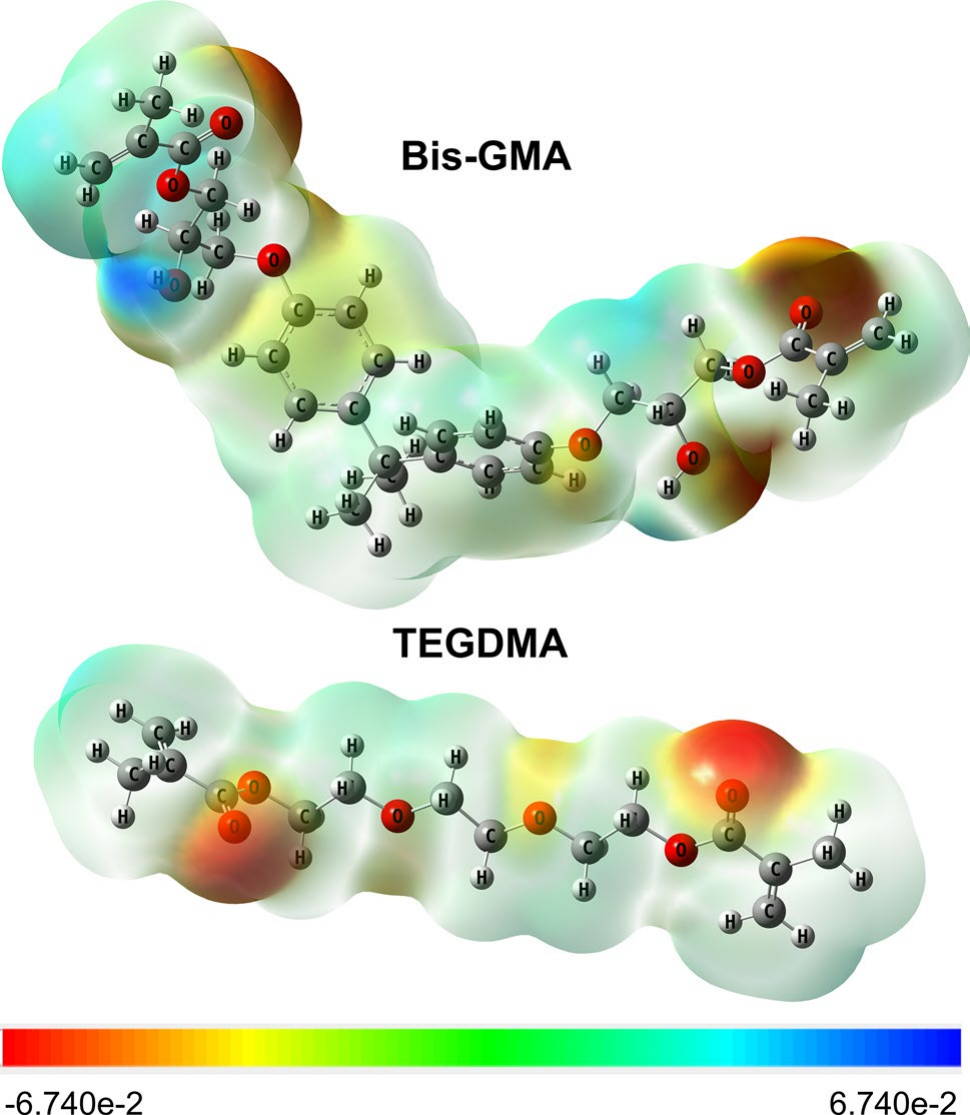
The optimized structures of the main components of the organic matrix of studied RBC with the marked electrostatic potential.

The predominant component of SE in the case of the studied composite is connected with dispersive interactions (γ^d^). The value of γ^d^ is attributed to the occurrence of van der Waals forces between all atoms. For specific interactions of a studied RBC, the values of γ^-^ are almost twice higher than the values of γ^+^. It means that the material acts more as an electron donor than electron acceptor. The explanation lies in the structure of its main components (Fig. 1).

The optimised Bis-GMA and TEGDMA structures with electrostatic potential acts as a simplified presentation of the electron donor sites, which are probably also observed in the polymeric forms of these monomers. The potential electron donor sites that are marked by red–orange colors in Fig. 1 are oxygens with free electron pairs, mainly ester oxygens with double bonds (=O) in both monomers, as well as hydroxyl oxygen (–OH) in Bis-GMA. The presence of these sites results in the ability of studied material to interact as electron donor rather than electron acceptor.

**Figure 2:**
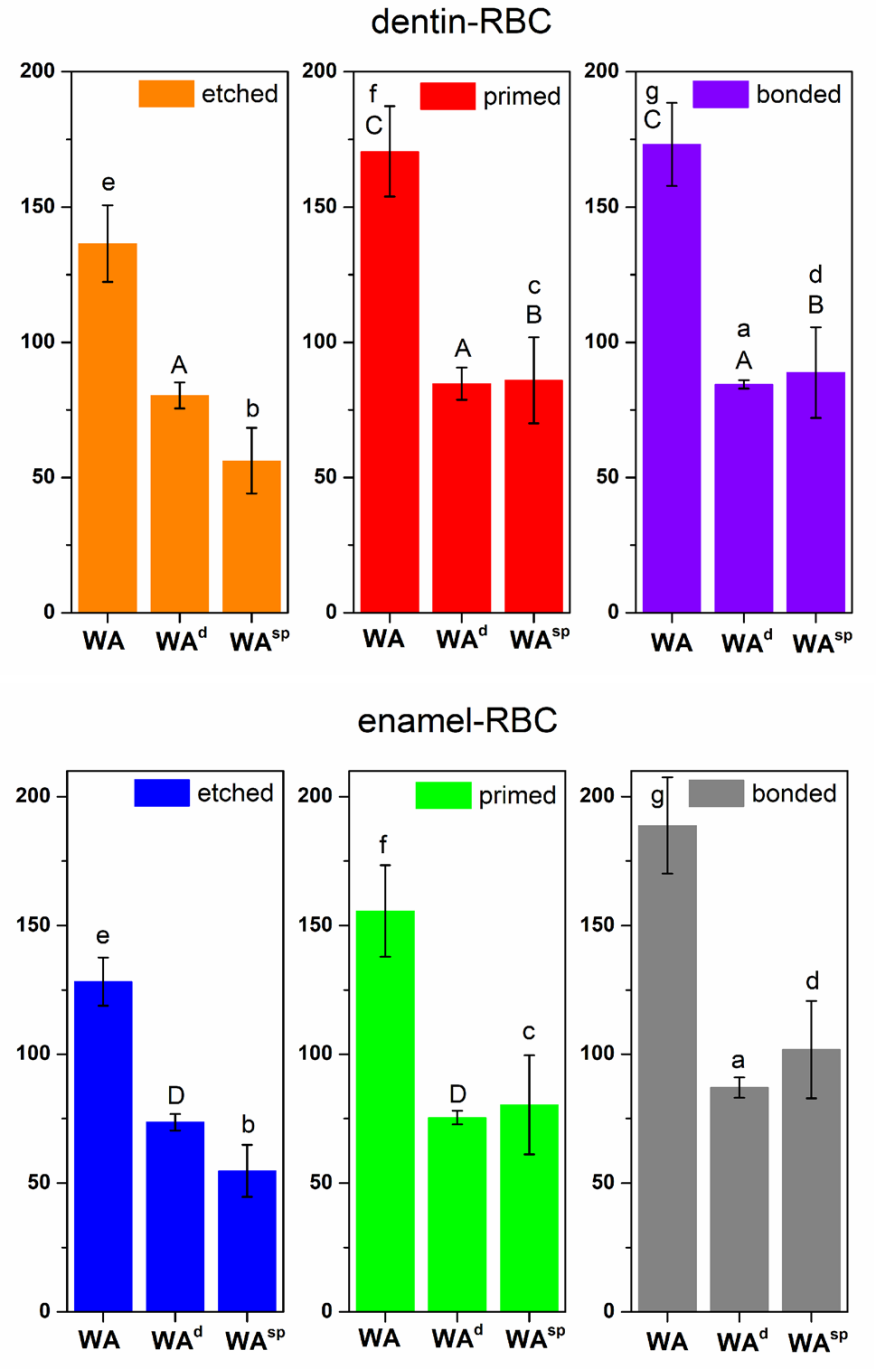
Mean values of WA and its dispersive (WA^d^), and specific (WA^sp^) components for all examined configurations [mJ·m^−2^]; the same capital letters mean no statistically significant differences (*p* > 0.05) in the values of each parameter between dentin or enamel samples after each step of preparation; the same lower cases mean no statistically significant differences (*p* > 0.05) in the values of each parameter between etched, primed or bonded dentin and enamel.

The values of dispersive and specific components of WA, as well as total WA values, calculated based on SE components of RBC and tooth hard tissues from^24^ are presented in Fig. 2.

According to the analysis of variance, there are no statistically significant differences (*p* > 0.05) in the values of the dispersive component of WA (WA^d^) between examined RBC and dentin independent on the stage of its preparation (A). It means that values of WA^d^ are the same for the following configurations: etched dentin-RBC, primed dentin-RBC and bonded dentin-RBC. Subsequent steps of preparation of dentin surface do not influence this tissue ability to interact by dispersive interactions with examined composite. The values of the specific component of WA (WA^sp^) increase (*p* < 0.05) after priming of dentin surface with no further statistically significant differences after application of the bonding agent (B). It means that the application of a primer to the etched dentin surface increases its ability to specific interactions with examined composite. It could be because new functional groups, which originate from the primer components appeared on the surface of primed dentin. The appearance of these groups was confirmed by the rich Raman scattering spectrum obtained on the surface of primed dentin, which was described in details in our previous paper^24^. Probably, since the surface of primed dentin is impregnated with a large number of groups able to specific interactions with examined composite, further application of bonding agent does not increase this total ability to interact on the specific way with RBC. Although the value of WA^sp^ does not change significantly after application of the adhesive, the active groups responsible for the magnitude of this parameter change. Active groups originating from primer react from one side with dentin (hydrophilic groups), while on the other side they can react with more hydrophobic compounds, for example, monomers from an adhesive or RBC. This ability to react is expressed as the value of a specific component of work of adhesion between the primed dentin and RBC (WA^sp^). However, the hydrophobic groups from primer react subsequently in the next step of dentin preparation with adhesive, while the adhesive introduces new groups able to bond with primer and with RBC. Therefore, the number of active sites remains in that case unchanged. The total value of work of adhesion (WA) show exactly the same statistical tendency as it was described for WA^sp^, i.e. the values of WA between dentin and examined RBC increase after priming of dentin (*p* < 0.05) with no further changes after application of bonding agent (C). It is caused by the fact that WA is calculated as the sum of dispersive (WA^d^) and specific (WA^sp^) components. Therefore, if the values of one parameter do not change (WA^d^) the tendency of WA changes is similar to the changes of WA^sp^ parameter.

Statistical analysis (one-way variance analysis) showed that the values of W^d^ between composite and enamel increase after application of bonding agent on the enamel structure (*p* < 0.05). It means that dispersive interactions between the examined composite and so-called bonded enamel are stronger than these observed between RBC and etched or primed enamel. No statistically significant difference in WA^d^ values in case of etched and primed enamel was observed (D). Statistically significant differences in values of WA^sp^ for examined composite and enamel are observed after each subsequent step of enamel preparation (*p* < 0.05). RBC and enamel are more capable of interacting together in a specific way after priming and bonding. It means that these both processes increase the values of a specific component of the work of adhesion (WA^sp^). The introduction of new active groups onto the enamel's surface with primer and adhesive components is responsible for this increase. In contrast to the primed and bonded dentin, the statistically significant difference between WA^sp^ values after priming and bonding of enamel is probably a result of a less complicated, more homogeneous structure of enamel^33^. Probably a lower number of primer's active groups were attached to the enamel's surface resulting in this slight but statistically significant difference in WA^sp^ value between primed enamel and bonded enamel. The same situation is observed in the case of total values of work of adhesion (WA), i.e. each subsequent step of preparation provides higher values of this parameter.

In our previous paper, it was stated that the surface activity expressed as SE parameters values equalise for enamel and dentin after etching and priming of their surface with subsequent much higher increase of enamel SE after application of adhesive (bonding agent) in comparison with bonded dentin^24^. It was hypothesised that it would reflect in different values of calculated work of adhesion between these tissues and an exemplary composite. To check this hypothesis Student’s t-tests in pairs were conducted. Each dentin-RBC configuration was compared with each corresponding enamel-RBC configuration to establish whether the statistically significant differences occur between the values of WA^d^, WA^sp^ and WA for etched/primed/bonded dentin-RBC and etched/primed/bonded enamel-RBC. Obtained results proved that this difference could be observed only in the case of the dispersive component of the work of adhesion (WA^d^) for etched and primed tissues. In both cases etched dentin-RBC and primed dentin-RBC are characterised by the higher WA^d^ values than etched enamel-RBC and primed enamel-RBC. However, after the whole process of tissues preparation (etching, priming, bonding) the values of this parameter for the configuration of RBC with both tissues (dentin and enamel) are the same (*p* < 0.05) (a). In the case of WA^sp^ and WA there are no statistically significant differences (*p* > 0.05) regardless of the type of tissue on each preparation step (b, c, d, e, f, g). It would mean that the ability of these both tissues, after the whole process of their surface preparation with the applied bonding system, to bond with composite should be the same.

**Figure 3:**
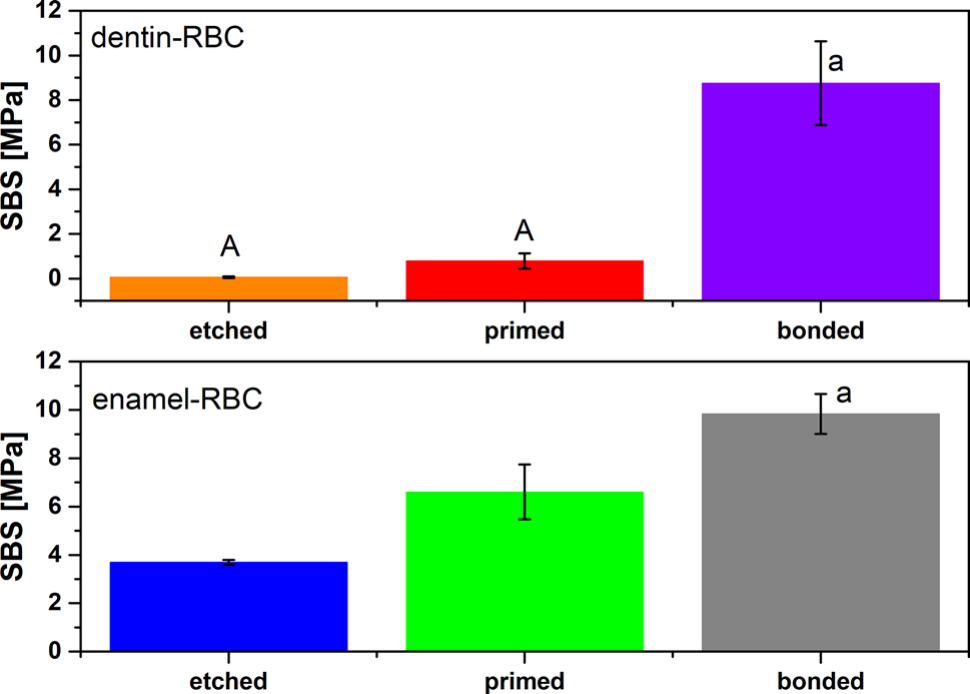
Mean values of SBS for all examined configurations; the same capital letters mean no statistically significant differences (*p* > 0.05) in the values of SBS between dentin or enamel samples after each step of preparation; the same lower cases mean no statistically significant differences (*p* > 0.05) in the values of SBS between etched, primed or bonded dentin and enamel.

Calculation of WA and its components (WA^d^ and WA^sp^) based on chromatographic data depends on the values of SE parameters of both solids to be bonded together (i.e. tooth hard tissues and restorative material). The values of all SE parameters of the examined composite are constant; thus, WA depends mainly on the values of SE parameters of dentin and enamel after each step of preparation. Etching, priming, and application of bonding agent result in the morphological and chemical changes of tissues surface^24^, what naturally impact its ability to interact with other materials (e.g. composites). The results of calculated WA parameters also confirmed it. Actual bond strength measured directly in the examined configurations employing mechanical testing is presented in Fig. 3.

According to the variance analysis, there are no statistically significant differences in values of SBS between etched dentin-RBC and primed dentin-RBC (*p* > 0.05) (A). Significantly higher values of SBS are observed in the case of dentin after the whole preparation procedure, i.e. after etching, priming and application of adhesive (bonded dentin-RBC) (*p* < 0.05). In the case of enamel, statistically significant differences in bond strength are observed after each subsequent step of the tissue preparation. Bonding of a composite to etched enamel is the weakest, while bond strength between this RBC and enamel after the whole preparation process (etching, priming, application of bonding agent) is the highest (*p* < 0.05).

**Figure 4:**
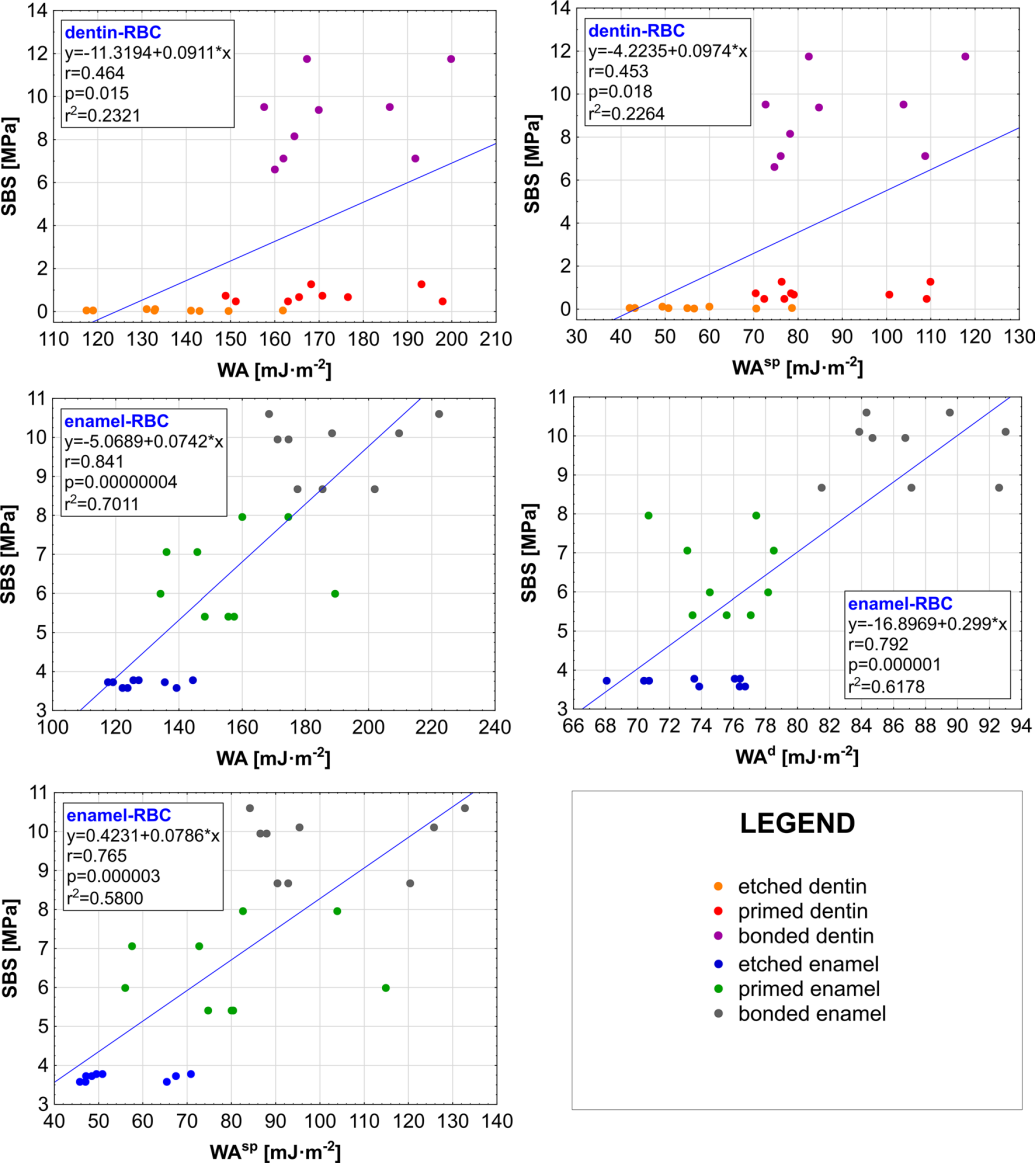
The results of correlation analysis for all statistically significant correlations (*p* < 0.05); r—correlation coefficient, *p*—the probability level; r^2^—coefficient of determination.

The differences in the ability to bond composites by dentin and enamel after each step of preparation were checked with a Student’s t-tests in pairs. Each dentin-RBC configuration was compared with each corresponding enamel-RBC configuration to establish whether the statistically significant differences occur between the values of SBS for etched/primed/bonded dentin-RBC and etched/primed/bonded enamel-RBC. In the case of etched and primed tissues (etched dentin and etched enamel), the composite is always bonded stronger to the enamel (higher values of SBS) (*p* < 0.05). The bond strength obtained after the application of bonding agent, i.e. after the whole preparation procedure, is the same for bonded dentin-RBC as for bonded enamel-RBC (*p* > 0.05) (a). It means that there are no statistical differences between bonding to dentin and bonding to enamel when these tissues are fully prepared. It is consistent with the results of the work of adhesion (WA) from IGC. Therefore, it seems that the results of both methods (IGC and SBS test) show some correlation. This thesis was subjected to correlation analysis of WA, WA^d^, WA^sp^, and SBS. The results of correlation analysis, regression equation, correlation coefficients (r), probability level (*p*), and coefficient of determination (r^2^) for all statistically significant correlations (*p* < 0.05) are presented in Fig. 4.

It can be observed that in the case of dentin, the values of SBS correlate positively with the values of a specific component of the work of adhesion (WA^sp^) and its total value (WA). The correlation coefficient is below 0.5, so both correlations should be considered as average; however, they exist. Moreover, these correlations are far from linear (low r^2^ values). In the case of enamel correlations between SBS and the values of all three WA parameters are observed. These correlations are positive and very high—r values higher than 0.75. Coefficients of determination also proved that these correlations are much closer to the linear model than it was observed in the case of dentin. The most important fact is that the research hypothesis cannot be rejected—there is a positive correlation between work of adhesion (WA) calculated based on chromatographic data from IGC experiments and shear bond strength (SBS) determined by the direct mechanical testing method. In the case of enamel, this correlation is very high (r = 0.841), while in the case of dentin, it was smaller (r = 0.464) but still statistically significant. It is probably because dentin, according to its more complex chemical composition and structure, requires more complicated preparation procedure than enamel. Enamel is composed of 96% of regularly organised hydroxyapatite in the form of enamel prisms, with only 3% of water and 1% of lipids and proteins. In comparison, dentin is less homogeneous tissue with 50% of hydroxyapatite, 30% of organic compounds, mainly collagen, and 20% of the plasma-like fluid. This tissue is composed of dentinal tubules with the differential amount and orientation^33^. Only the application of bonding agent to dentin, so conducting the whole preparation procedure, resulted in the statistically significant increase of SBS values. It means that in contrast to the theoretical values obtained based on IGC, the actual bond strength was much lower before the application of bonding agent on the dentin surface, close to zero. Therefore, the correlation between WA and SBS for dentin-RBC connections is average. Moreover, dentin is considered to be much more complicated substrate than enamel^34^. The weaker correlation of SBS values with WA values in dentin substrate can also result from the higher probability of mixed or cohesive modes of failure in the analysed junction when compared with the enamel substrate, in which the adhesive failure is more probable during its higher strength. However, it should be remembered that the standard clinical procedure always assumes the execution of the whole preparation procedure. In the case of other less complicated tissue, i.e. enamel, SBS results are well covered by IGC results what undoubtedly prove the applicability of IGC in such experiments.

## Conclusions

The research hypothesis was not rejected. There is a correlation between the values of two parameters describing directly (SBS) and indirectly (WA) the bonding between tooth hard tissues and composite restorative material. The observed correlation was stronger in the case of enamel-RBC combinations than dentin-RBC combinations what is probably caused by the more complicated chemical structure of dentin and the fact that the comparison was conducted for each subsequent step of these tissues surface preparation, as well as probable differences in failure modes. This study presents preliminary results and should be extended to further research with other restorative materials to bring more detailed knowledge into the field of the bond strength examination employing IGC. However, despite all limitations of this study, it should be recognised that inverse gas chromatography is a powerful perspective tool in the examination of bond strength between tooth hard tissues and potential dental materials without using a large number of healthy teeth.
